# Overweight and obesity among adults in Germany

**DOI:** 10.17886/RKI-GBE-2017-038

**Published:** 2017-06-14

**Authors:** Anja Schienkiewitz, Gert B. M. Mensink, Ronny Kuhnert, Cornelia Lange

**Affiliations:** Robert Koch Institute, Department for Epidemiology and Health Monitoring, Berlin, Germany

**Keywords:** OVERWEIGHT, OBESITY, SELF-REPORTING, HEALTH MONITORING, GERMANY

## Abstract

Body weight and height, as well as associated indicators like overweight and obesity, are widespread factors used to describe the health of a population. Over the past decades, the prevalence of overweight and obesity has increased worldwide and has reached significant public health relevance. According to self-reported data on body weight and body height in the GEDA 2014/2015-EHIS study, 54.0% of adults in Germany are overweight or obese (defined as having a body mass index – BMI – of 25 kg/m^2^ or higher). Men are more often affected by overweight than women, with 43.3% of men having a BMI between 25 kg/m^2^ and 30 kg/m^2^, compared to women (28.8%). In Germany, the prevalence of obesity (BMI greater or equal to 30 kg/m^2^) is 18.1%; there is no significant difference between women and men. The prevalence of overweight, including obesity, is higher among women and men with increasing age. Although the prevalence of overweight, including obesity, has remained at a high level in recent years, the prevalence of obesity has increased compared to the GEDA 2010 study.

## Introduction

Persons are defined as overweight if their body weight exceeds a certain level for a given body height. Excessive overweight is referred to as obesity and classified as a disease by the World Health Organization (WHO) [1]. Obesity is a risk factor linked to chronic diseases such as type 2 diabetes mellitus [2], cardiovascular diseases [3] and some types of cancer [4]. It is also associated with a higher risk of premature death [5, 6]. Finally, obesity and the associated comorbidities are a major challenge to the health system and present an important public health problem not only in Germany but also worldwide.

Against this background, the WHO developed the Global Action Plan for the Prevention and Control of Non-communicable Diseases 2013-2020. One of the 9 voluntary global non-communicable diseases targets addresses the prevalence of obesity. The rise in diabetes and obesity prevalence should be halted until 2025 on the 2010 levels [7]. In accordance to the WHO Global Action Plan, the revision of the Sustainable Development Strategy 2016 of the Federal Government of Germany has the target that until 2030 the proportion of people with obesity in Germany does no longer increase [8].

## Indicator

Body Mass Index (BMI) is the most commonly used measure to define overweight and obesity. It is calculated as the ratio of a person’s body weight to the square of his body height (kg/m^2^); it is thus relatively easy to calculate and to use as a reference measure for individuals as well as study populations. The BMI is no direct measure of body fat as it cannot distinguish between body fat and muscle mass. However, research has shown that at the group level BMI shows a high correlation with direct measurements used to determine body fat. A high BMI can therefore act as an indicator of a high level of body fat. According to the WHO classification system, adults with a BMI of less than 18.5 kg/m^2^ are considered to be underweight. A BMI between 18.5 kg/m^2^ and less than 25 kg/m^2^ is defined as normal weight, a BMI between 25 kg/m^2^ and under 30 kg/m^2^ as overweight and a BMI of 30 kg/m^2^ or more as obese [1].

**GEDA 2014/2015-EHIS**

**Data holder:** Robert Koch Institute

**Aims:** To provide reliable informa tion about the population’s health status, health-related behaviour and health care in Germany, with the possibility of a European comparison

**Method:** Questionnaires completed on paper or online

**Population:** People aged 18 years and above with permanent residency in Germany

**Sampling:** Registry office sample; randomly selected individuals from 301 communities in Germany were invited to participate

**Participants:** 24,016 people (13,144 women; 10,872 men)

**Response rate:** 26.9%

**Study period:** November 2014 - July 2015

**Data protection:** This study was undertaken in strict accordance with the data protection regulations set out in the German Federal Data Protection Act and was approved by the German Federal Commissioner for Data Protection and Freedom of Information. Participation in the study was voluntary. The participants were fully informed about the study’s aims and content, and about data protection. All participants provided written informed consent.

More information in German is available at www.geda-studie.de

In order to calculate BMI, studies collect data on body weight and body height either through direct measurement or self-reporting. Self-reporting often leads to underestimated body weights and overestimated body heights compared with directly measured values. A BMI calculated through self-reported information thus tends to be lower than those gained through direct measurements [9]. The prevalence presented here based on the German Health Update (GEDA) study series used self-reported data. Therefore, the prevalence observed in this study differs from the prevalence calculated using data from direct measurements gathered for the National Health Interview and Examination Surveys conducted by the Robert Koch Institute, including the 1998 German National Health Interview and Examination Survey (GNHIES98) and the German Health Interview and Examination Survey for Adults (DEGS1) [10].According to DEGS1 the prevalence of obesity in the age group 18 and 79 years is 23.9% among women and 23.3% among men. For the direct comparison of obesity prevalence from different data sources, such as DEGS1 and GEDA 2014/2015-EHIS, it should be taken into account that the obesity prevalence from self-reported data is lower. Furthermore, comparisons from GEDA 2014/2015-EHIS with previous GEDA waves need to take into account the fact that sampling methods and types of questionnaire (self-administered questionnaire versus telephone interview) have been changed.

As part of the GEDA 2014/2015-EHIS study, respondents were asked: ‘How tall are you without shoes (in cm)?’. The question on body weight was: ‘How much do you weigh without clothes and shoes (in kg)? Pregnant women should provide their weight before they became pregnant.’

The tables present the prevalence of underweight, normal weight, overweight, obesity as well as overweight including obesity among the German population aged 18 years and older. The results are stratified according to gender, age and level of education, and for obesity additionally, by gender and federal state.

The analyses are based on data from 23,791 participants aged 18 years and older (13,006 women and 10,785 men) with valid data on body weight and height. The calculations were carried out using a weighting factor that corrects for deviations within the sample from the German population (as of 31 December 2014) with regard to gender, age, district type and education. The district type accounts for the degree of urbanisation and reflects the regional distribution in Germany. The International Standard Classification for Education (ISCED) was used to ensure that the responses provided on educational levels were comparable [11]. A detailed description of the methodology applied in the GEDA 2014/2015-EHIS study can be found in the article German Health Update – New data for Germany and Europe in issue 1/2017 of the Journal of Health Monitoring.

## Results and discussion

The results of the GEDA 2014/2015-EHIS study indicate that 46.7% of women and 61.6% of men in Germany have a BMI of more than 25 kg/m^2^ and are thus overweight or obese. 28.8% of women and 43.3% of men have a BMI between 25 kg/m^2^ and less than 30 kg/m^2^, and 18.1% of adults are obese (Table 1 and Table 2). Overall, the prevalence of overweight, including obesity, has remained high in recent years. In 2012, 45.8% of women and 59.7% of men were overweight or obese [12]. The GEDA 2014/2015-EHIS data show no significant difference between women and men, with the preva lence increasing by two percentage points compared to 2010 [12].

The current prevalence and trends in obesity is similar as observed in the German Microcensus which also uses self-reported data on body weight and height. In 2013, 14.3% of women and 17.1% of men were obese [13]. This is an increase of 3.3 percentage points among women and 5.0 percentage points among men compared to earlier assessments of BMI in the German Microcensus conducted in 1999. At that time, the obesity prevalence was 11.5% among adults (11.0% of women and 12.1% of men) [14]. Current results from the GEDA 2014/2015-EHIS study largely confirm the trends observed from the German Microcensus on trends in obesity prevalence.

The prevalence of overweight, including obesity, rises with increasing age among both women and men. This is also consistent with results of previous surveys [12]. Over time, the prevalence of obesity has increased significantly, particularly among younger age groups. Between 2010 and 2014/2015, the prevalence of obesity among 18- to 29-year-olds increased from 5.5% to 9.7% among women and from 5.4% to 8.9% among men. No further increase was observed during this period among adults aged 65 years and older. This trend also corresponds with results of the National Health Interview and Examination Surveys conducted by the RKI [10]. Furthermore, over 80% of adults with obesity remain obese after 10 years [15] and thus have an increased risk of various health problems and chronic diseases.

The prevalence of obesity also varies according to certain social characteristics: obesity is more common among people with a low level of education compared to those with high education levels. The difference regarding education level is observed among women of all age groups except the 65 year-olds and older (Table 1). Among men, the differences in educational level appear only in the age group 45 years and older (Table 2).

**Figure 1:**
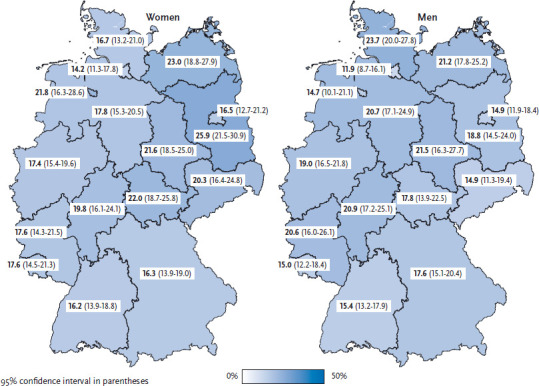
Obesity according to gender and German federal state (n=13,006 women; n=10,785 men) Source: GEDA 2014/2015-EHIS

Compared to the average obesity prevalence over all federal states, the highest prevalence estimates were observed among women in Brandenburg and Mecklenburg-West Pomerania and among men in Mecklenburg-West Pomerania and Schleswig-Holstein. In contrast, women in Hamburg and Baden-Württemberg and men in Hamburg have significantly lower prevalence estimates (Figure 1). Data from the German Microcensus also allow a detailed analysis of the obesity prevalence at regional levels. For all reported years, there is a gradient from the northeast to the southwest, with higher obesity prevalence in Brandenburg and Mecklenburg-West Pomerania and a lower prevalence in Baden-Württemberg [16]. In addition, the German Micro-census can also be used to provide estimates for regions within federal states. These estimates show that the prevalence of obesity among the population within the federal states differs considerably [17].

In summary, the upwards trend of obesity prevalence is continuing. This is in contrast to the targets of the Global Action Plan for the Prevention and Control of Non-communicable Diseases 2013-2020 and the Sustainable Development Strategy 2016 of the Federal Government, both of which aim to halt the rise in obesity prevalence [7, 8].

## Key statements

- 47% of women and 62% of men in Germany are overweight or obese; 18% of adults are obese.
- The prevalence of overweight, including obesity, is higher among both women and men with age.
- The prevalence of obesity has increased compared with previous surveys. A significant increase is particularly evident among younger age groups.

## Figures and Tables

**Table 1:** Underweight, normal weight, overweight and obesity among women according to age and educational status (n=13,006) Source: GEDA 2014/2015-EHIS

Women	Underweight		Normal weight		Overweight		Obesity		Overweight including obesity*	
	%	(95% CI)	%	(95% CI)	%	(95% CI)	%	(95% CI)	%	(95% CI)
**Women total**	**2.9**	**(2.5-3.3)**	**50.4**	**(49.3-51.6)**	**28.8**	**(27.8-29.8)**	**18.0**	**(17.1-18.9)**	**46.7**	**(45.6-47.9)**
**18-29 Years**	7.5	(6.2-9.0)	66.4	(63.7-68.9)	16.5	(14.5-18.7)	9.7	(8.3-11.3)	26.2	(23.8-28.6)
Low education	10.9	(7.4-15.8)	56.7	(50.6-62.6)	19.3	(14.9-24.6)	13.1	(9.3-18.1)	32.4	(26.9-38.4)
Medium education	6.5	(5.2-8.2)	67.5	(64.1-70.8)	16.3	(13.8-19.3)	9.6	(7.9-11.6)	25.9	(23.0-29.1)
High education	5.4	(3.5-8.3)	77.3	(72.6-81.4)	12.3	(9.5-15.7)	5.0	(3.3-7.5)	17.3	(13.9-21.3)
**30-44 Years**	2.4	(1.8-3.1)	56.1	(53.7-58.5)	24.2	(22.2-26.3)	17.3	(15.4-19.4)	41.5	(39.1-43.9)
Low education	2.1	(0.8-5.8)	34.5	(28.4-41.1)	31.9	(25.7-38.8)	31.5	(24.7-39.2)	63.4	(56.6-69.6)
Medium education	2.1	(1.4-3.0)	55.2	(52.2-58.2)	25.3	(22.8-28.0)	17.4	(15.2-19.9)	42.7	(39.6-45.8)
High education	3.0	(2.0-4.6)	70.6	(67.5-73.5)	17.3	(15.0-19.9)	9.1	(7.2-11.4)	26.4	(23.7-29.3)
**45-64 Years**	1.6	(1.2-2.0)	48.3	(46.6-50.0)	30.5	(28.9-32.2)	19.6	(18.3-21.1)	50.1	(48.4-51.9)
Low education	1.4	(0.7-2.9)	43.3	(39.0-47.8)	33.1	(28.9-37.6)	22.2	(18.7-26.0)	55.3	(50.9-59.5)
Medium education	1.6	(1.2-2.1)	46.9	(44.8-49.1)	30.4	(28.4-32.5)	21.1	(19.3-22.9)	51.5	(49.3-53.7)
High education	1.7	(1.1-2.6)	57.4	(54.4-60.2)	28.3	(25.8-30.9)	12.7	(10.9-14.7)	41.0	(38.1-43.9)
**≥ 65 Years**	2.2	(1.5-3.1)	38.9	(36.6-41.2)	37.6	(35.5-39.8)	21.3	(19.4-23.3)	58.9	(56.5-61.3)
Low education	2.5	(1.5-4.2)	37.7	(34.1-41.5)	37.6	(34.1-41.3)	22.2	(19.1-25.5)	59.8	(55.9-63.6)
Medium education	2.2	(1.4-3.5)	38.2	(34.9-41.6)	38.8	(35.8-41.9)	20.8	(18.3-23.5)	59.6	(56.0-63.0)
High education	0.8	(0.3-2.2)	46.5	(41.3-51.8)	32.0	(27.4-37.1)	20.6	(16.2-25.8)	52.7	(47.4-57.9)
**Total (women and men)**	**1.8**	**(1.6-2.1)**	**44.1**	**(43.2-45.1)**	**35.9**	**(35.1-36.7)**	**18.1**	**(17.4-18.9)**	**54.0**	**(53.1-54.9)**

CI=confidence interval

* Deviations in the prevalence of ‘overweight including obesity’ from the sum of the prevalence of ‘overweight’ and ‘obesity’ are due to rounding

**Table 2:** Underweight, normal weight, overweight and obesity among men according to age and educational status (n=10,785) Source: GEDA 2014/2015-EHIS

Men	Underweight		Normal weight		Overweight		Obesity		Overweight including obesity*	
	%	(95% CI)	%	(95% CI)	%	(95% CI)	%	(95% CI)	%	(95% CI)
**Men total**	**0.8**	**(0.6-1.0)**	**37.6**	**(36.3-38.9)**	**43.3**	**(42.1-44.5)**	**18.3**	**(17.3-19.4)**	**61.6**	**(60.3-62.9)**
**18-29 Years**	3.2	(2.4-4.4)	62.8	(59.7-65.8)	25.1	(22.3-28.0)	8.9	(7.2-10.8)	33.9	(31.0-37.0)
Low education	6.3	(3.9-10.3)	60.6	(53.8-66.9)	23.9	(18.6-30.3)	9.2	(5.8-14.3)	33.1	(27.1-39.7)
Medium education	2.3	(1.5-3.6)	61.5	(57.6-65.3)	26.2	(22.9-29.8)	10.0	(7.8-12.6)	36.2	(32.4-40.1)
High education	1.4	(0.4-4.8)	73.2	(67.9-77.9)	21.8	(17.3-27.1)	3.6	(2.0-6.5)	25.4	(20.7-30.8)
**30-44 Years**	0.1	(0.1-0.4)	39.9	(37.2-42.6)	42.6	(40.0-45.3)	17.3	(15.3-19.6)	60.0	(57.2-62.7)
Low education	0.3	(0.0-2.3)	37.1	(28.9-46.2)	45.5	(36.8-54.3)	17.1	(11.3-25.0)	62.5	(53.4-70.8)
Medium education	0.0	(0.0-0.2)	36.6	(33.3-40.0)	43.0	(39.6-46.5)	20.4	(17.5-23.5)	63.4	(60.0-66.7)
High education	0.3	(0.1-0.8)	46.8	(43.0-50.8)	41.1	(37.4-44.8)	11.8	(9.6-14.5)	52.9	(49.0-56.8)
**45-64 Years**	0.2	(0.1-0.4)	29.7	(28.0-31.5)	48.2	(46.3-50.1)	21.9	(20.3-23.6)	70.1	(68.3-71.7)
Low education	0.3	(0.0-2.2)	27.3	(22.4-32.8)	46.8	(41.3-52.4)	25.6	(21.3-30.4)	72.4	(67.0-77.3)
Medium education	0.2	(0.1-0.6)	28.0	(25.9-30.4)	47.9	(45.3-50.6)	23.8	(21.4-26.3)	71.7	(69.4-73.9)
High education	0.1	(0.0-0.5)	33.5	(30.9-36.1)	49.3	(46.7-51.9)	17.2	(15.2-19.3)	66.4	(63.8-69.0)
**≥ 65 Years**	0.5	(0.3-0.9)	28.2	(26.3-30.3)	50.4	(48.2-52.7)	20.9	(19.0-22.8)	71.3	(69.2-73.2)
Low education	0.6	(0.1-3.6)	25.5	(21.0-30.6)	49.2	(44.0-54.4)	24.7	(20.5-29.5)	73.9	(68.9-78.3)
Medium education	0.4	(0.2-1.0)	26.9	(24.0-30.1)	51.1	(47.4-54.8)	21.5	(18.8-24.5)	72.7	(69.5-75.6)
High education	0.5	(0.2-1.2)	32.0	(29.1-35.1)	49.9	(46.8-53.0)	17.6	(15.1-20.4)	67.5	(64.5-70.3)
**Total (women and men)**	**1.8**	**(1.6-2.1)**	**44.1**	**(43.2-45.1)**	**35.9**	**(35.1-36.7)**	**18.1**	**(17.4-18.9)**	**54.0**	**(53.1-54.9)**

CI=confidence interval

* Deviations in the prevalence of ‘overweight including obesity’ from the sum of the prevalence of ‘overweight’ and ‘obesity’ are due to rounding
